# Massive pulmonary embolism and intra-cardiac thrombus requiring systemic thrombolysis 9-hours post emergency laparotomy

**DOI:** 10.1093/jscr/rjac528

**Published:** 2022-11-28

**Authors:** Andrew Stafford Beatty, Fraser Hugh Simpson, Manju D Chandrasegaram

**Affiliations:** Department of General Surgery, The Prince Charles Hospital, Brisbane, Queensland, Australia; Northside Clinical School, School of Medicine, The University of Queensland, Brisbane, Queensland, Australia; Department of General Surgery, The Prince Charles Hospital, Brisbane, Queensland, Australia; Northside Clinical School, School of Medicine, The University of Queensland, Brisbane, Queensland, Australia; Department of General Surgery, The Prince Charles Hospital, Brisbane, Queensland, Australia; Northside Clinical School, School of Medicine, The University of Queensland, Brisbane, Queensland, Australia

## Abstract

The link between abdominal surgery and venous thromboembolism (VTE) has been well established with recent evidence exploring the optimal VTE risk reducing strategy. However, despite these strategies pulmonary embolisms (PEs) do occur, which in the immediate post-operative setting creates a dilemma; to treat the VTE with anticoagulation but balance against the risk of hemorrhage. Treatment guidelines often do not include post-operative patients leaving the decision up to the treating physician to weigh the relative risks on an individual basis. We present a 59-year-old lady who developed a life-threatening submassive PE within 9 h of an emergency laparotomy for a perforated rectal cancer. She was treated with systemic thrombolysis after alternative interventions had been excluded. She responded well to therapy with no major bleeding. She was successfully discharged home after a short period of inpatient rehabilitation.

## INTRODUCTION

Surgery and malignancy are two risk factors for the development of a venous thromboembolism (VTE) [1, 2]. Studies have shown that pulmonary embolism (PE) to be the third most common cause of death for hospitalized patients [3]. The origins of systemic thrombolysis date back to 1933 [4] however since its introduction the major complication continues to be bleeding. Despite the risk of VTE associated with gastrointestinal surgery there is little evidence to guide post-operative thrombolysis due to this bleeding risk. Herein we present the case of a massive pulmonary embolism within 9 h of major abdominal resection successfully treated with systemic thrombolysis.

## CASE REPORT

A 59-year-old lady presented to the emergency department with acute severe abdominal pain having recently been diagnosed with locally advanced metastatic rectal adenocarcinoma awaiting commencement of neoadjuvant chemotherapy. Her past medical history included obesity with body mass index of 36.8 and osteoarthritis. She was functionally independent and took no regular medications.

On arrival to the emergency department, she was febrile, tachycardiac and had significant analgesic requirements. She proceeded to have a computed tomography (CT) scan of her abdomen and pelvis. This demonstrated a sigmoid perforation proximal to the rectal mass with significant fecal peritoneal contamination.

She was consented and proceeded to an emergency laparotomy. The initial consideration was to perform en-bloc resection but given the technical complexity and the patient’s rising inotropic requirements decision was made to perform a damage control procedure with washout, resection of the perforation and a planned relook after stabilization in the ICU. A laparostomy was performed and she was transferred to the ICU.

By morning her hemodynamics had improved and she returned to theatre. The rectal cancer was resected, an end colostomy was created and her abdomen successfully closed. By the end of the case the patient’s hemodynamics continued to improve with reducing inotropic requirements and improving acidosis. She returned to the ICU intubated and ventilated.

Eight hours post procedure there was a sudden rapid decrease in systolic blood pressure to 90 mmHg and oxygen saturations to 80%. She was developing rapidly increasing inotropic requirements. An urgent bedside transthoracic echocardiogram was performed, which demonstrated a collapsed left ventricle, right ventricular dilatation and a visible clot in transit between the right atrium and ventricle. During the echocardiogram the visualized clot disappeared coinciding with a rapid decline in oxygenation with unrecordable oxygen saturations and end tidal CO2.

Several options including thrombectomy and catheter-directed thrombolysis were considered however following discussion with the respective teams she was deemed too unstable for transfer. The more immediate option of systemic thrombolysis was considered, but the main concern was the potential risk for major catastrophic bleeding following her major abdominal surgery. Following intense multidisciplinary discussions, the consensus to offer systemic thrombolysis was reached with the acceptance for relook laparotomy should significant bleeding occur. Consent was obtained from the patient’s children as joint statutory health attorneys.

Following the administration of 100-mg alteplase inotropic requirement started to decrease within 4 h. Repeat echocardiogram revealed no residual atrial thrombus. Over the next 5 days she responded well and was successfully weaned off all inotropic support and extubated. She was successfully discharged to the ward on post-operative Day 10. Following inpatient rehabilitation, she was successfully discharged home on post-operative Day 18 with mild deconditioning but no neurological deficiencies. She was managed in the outpatient setting with no further readmissions.

She is now 9 months post her resection and has undergone adjuvant chemotherapy. Her surveillance imaging has shown a reduction in her liver metastases with no evidence of recurrence. She is functionally independent and has returned to part-time employment.

## DISCUSSION

VTE prophylaxis is a key element in the care plan of any postoperative patient be they elective or emergent. Post-operative VTE incidence has been estimated at 6% with associated mortality as high as 10% in some studies [1, 5, 6]. For those patients who develop a VTE international guidelines favor low-molecular weight heparin (LMWH) as the initial treatment however, their risk of bleeding is 2–6-fold higher than their non-cancer counterparts [7]. Sadly, these guidelines do not address the immediate post-operative period, nor do they cover submassive PEs.

Systemic thrombolysis in patients with submassive PE has been extensively investigated. A meta-analysis by Chatterjee *et al*. reviewed 1061 patients receiving thrombolysis against 1054 who received only anticoagulation. They found a reduction in mortality but with a significant increase in bleeding, 9.24% against 3.42% [8]. This was supported by Riva *et al*. who conducted a 12 meta-analysis review where they found thrombolysis reduced all-cause mortality by a median of 1.55% [9].

To reduce the risk of bleeding came the emersion of catheter-directed therapies offering a lower dose of thrombolytics but delivered directly to the thrombus. The ULTIMA trial randomized 59 patients and found a more rapid improvement of the RV/LV ratio at 48 h. They reported no major bleeds in either arm [10]. The SEATTLE II trial enrolled 150 patient and confirmed these findings with rapid reduction in RV/LV but reported a higher bleeding risk of 10%, the researchers explained this in the studies differing definitions of bleeding [11]. The PERFECT registry of 101 patients has found catheter direct thrombolysis reduced pulmonary arterial pressure and without any major bleeds [12].

Surgical embolectomy via extracorporeal membrane oxygenation and pulmonary thrombarterectomy is another consideration. Historically this approach was not considered however recent data has suggested there is increasing survivability from this approach. A single Massachusetts institution published data from 115 patients who underwent surgical embolectomy, and their data showed a 1-year survival rate of 80.4% with a surgical mortality of 3.6% [13]. Regardless this approach requires rapid access to a cardiothoracic service with appropriate support. Rali and Criner recommend this option be reserved as a last resort [14].

## CONCLUSION

This case reflects a difficult scenario; indeed, it is the first case report we can find of systemic thrombolysis being administered so close to major abdominal resection. The authors accept the bleeding risk but given the lack of alternatives in a rapidly deteriorating patient palliation was the sole remaining consideration.

## References

[1] Geerts WH , Heit JA, Clagett GP, Pineo GF, Colwell CW, Anderson Jr FA, et al. Prevention of venous thromboembolism. Chest 2001;119:132–75. 10.1378/chest.119.1_suppl.132s 11157647

[2] Heit JA , Silverstein MD, Dn M, Petterson TM, O'Fallon WM, Melton LJ 3rd. Risk factors for deep vein thrombosis and pulmonary embolism: a population-based case-control study. Arch Intern Med 2000;160:809–15. 10737280 10.1001/archinte.160.6.809

[3] Tapson VF , Sterling K, Jones N, Elder M, Tripathy U, Brower J, et al. Acute pulmonary embolism. N Engl J Med 2008;358:1037–52. 18322285 10.1056/NEJMra072753

[4] Ouriel K . A history of thrombolytic therapy. J Endovasc Ther 2004;11:128–33. 10.1177/15266028040110S621 15760256

[5] Wu C , Alotaibi GS, Alsaleh K, Linkins L-A, McMurtry MS. Case Fatality of recurrent venous thromboembolism and major bleeding associated with aspirin, warfarin ad direct oral anticoagulants for secondary prevention. Thromb Res 2015;135:243–8. 25488466 10.1016/j.thromres.2014.10.033

[6] Leeds IL , Canner JK, DiBrito SR, Safar B. Justifying total costs of extended venothromboembolism prophylaxis after colorectal cancer surgery. J Gastrointest Surg 2020;24:677–87. 30945086 10.1007/s11605-019-04206-z PMC6776724

[7] Farge D , Debourdeau P, Beckers M, Baglin C, Bauersachs RM, Brenner B, et al. International clinical practice guidelines for the treatment and prophylaxis of venous thromboembolism in patients with cancer. J Thromb Haemost 2013;11:56–70. 23217107 10.1111/jth.12070

[8] Chatterjee S , Charkarborty A, Weinberg I, Kadakia M, Wilensky RL, Sardar P, et al. Thrombolysis for pulmonary embolism and risk of all-cause mortality, major bleeding and intracranial haemorrhage: a meta-analysis. JAMA 2014;311:2414–21. 24938564 10.1001/jama.2014.5990

[9] Riva N , Puljak L, Moja L, Ageno W, Schünemann H, Magrini N, et al. Multiple overlapping systematic reviews facilitate the origin of disputes: the case of thrombolytic therapy for pulmonary embolism. J Clin Epidemiol 2018;97:1–13. 29175415 10.1016/j.jclinepi.2017.11.012

[10] Kucher N , Boekstegers P, Müller OJ, Kupatt C, Beyer-Westendorf J, Heitzer T, et al. Randomized, controlled trial of ultrasound-assisted catheter-directed thrombolysis for acute intermediate-risk pulmonary embolism. Circulation 2014;129:479–86. 24226805 10.1161/CIRCULATIONAHA.113.005544

[11] Piazza G , Hohlfelder B, Jaff MR, Ouriel K, Engelhardt TC, Sterling KM, et al. SEATTLE II Investigators. A prospective, single-arm, multicentre trial of ultrasound-facilitated, catheter-directed, low-dose fibrinolysis for acute massive and sub-massive pulmonary embolism: the SEATTLE II study. JACC Cardiovasc Interv 2015;8:1382–92. 26315743 10.1016/j.jcin.2015.04.020

[12] Kuo WT , Banerjee A, Kim PS, DeMarco FJ Jr, Levy JR, Facchini FR, et al. Pulmonary embolism response to fragmentation, embolectomy and catheter thrombolysis (PERFECT): initial results from a prospective multicentre registry. Chest 2015;148:667–73. 25856269 10.1378/chest.15-0119

[13] Neely RC , Byrne JG, Gosev I, Cohn LH, Javed Q, Rawn JD, et al. Surgical embolectomy for acuet massive and sub-massive pulmonary embolism in a series of 115 patients. Ann Thorac Surg 2015;100:1245–52. 26165484 10.1016/j.athoracsur.2015.03.111

[14] Rail PM , Criner GJ. Submassive pulmonary embolism. Am J Respir Crit Care Med 2018;198:588–98. 29672125 10.1164/rccm.201711-2302CI

